# Computational studies of intermolecular interactions in aqueous solutions of poly(vinylmethylether)

**DOI:** 10.1007/s00894-014-2529-5

**Published:** 2014-11-26

**Authors:** J. Saramak, K. Halagan, M. Kozanecki, P. Polanowski

**Affiliations:** Department of Molecular Physics, Faculty of Chemistry, Lodz University of Technology, Zeromskiego 116, 90-924 Lodz, Poland

**Keywords:** Cooperative dynamics, Dynamic lattice liquid model, Graining procedure, Monte Carlo methods, Poly(vinylmethylether), Quantum calculations

## Abstract

Thermo-responsive materials, such as poly(vinylmethylether) (PVME), attract a common attention because of their unique physical properties resulted from metastable equilibrium between various types of interactions. In this work Monte Carlo (MC) and quantum-mechanical (QM) methods were used to study excluded volume and electrostatic interactions respectively. The graining procedure of PVME-water system was proposed. Its implementation to MC calculations allowed to distinguish how two water fractions differ on dynamics. The QM calculations showed that the formation of cyclic clusters leads to the lengthening of the hydrogen bonds and consequently to higher energies in comparison to linear forms, which is crucial looking at an application of QM results to MC calculation considering thermal interactions.

## Introduction

Polymer stimuli responsive hydrogels attract common attention because of both unique physico-chemical properties and many potential applications in micro electro-mechanical or biomedical systems for example: micro-valves [1], artificial muscles [2], sensors and chemical indicators [3], drug delivery systems [4, 5], selective regenerable membranes [6], lenses with changeable focal length [6], and others. Anomalous behaviors of stimuli responsive hydrogels result from a metastable equilibrium between polymer-water, water-water, and polymer-polymer interactions [7].

**Fig. 1:**
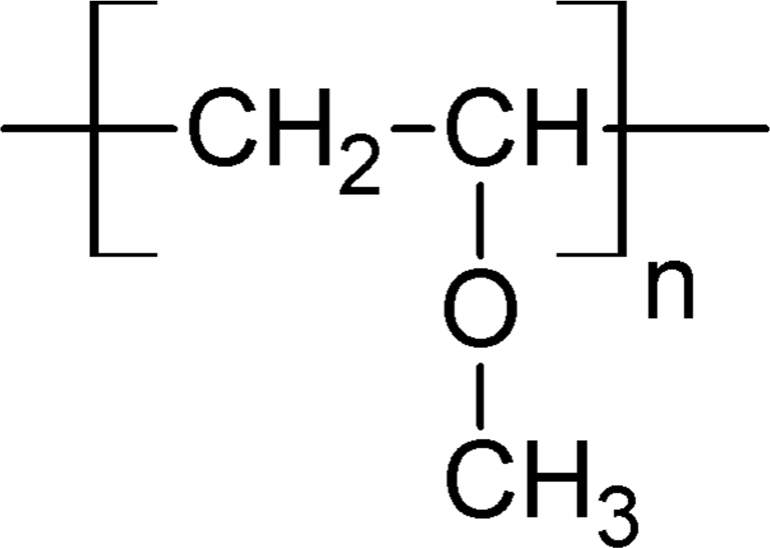
Chemical structure of PVME

Poly(vinylmethylether) (PVME) is a perfect model of stimuli responsive polymer because of simple chemical structure (see Fig. 1) with only one hydrophilic center (oxygen atom); PVME is a thermo-sensitive polymer with restricted miscibility in water to the temperature range below c.a. 37 °C [4, 8, 9]. If the temperature of the system exceeds this threshold, called lower critical solution temperature (LCST), the hydrogen bonds between water and polymer abruptly break and the phase separation occurs. In a case of crosslinked systems, such as hydrogels, this process is called volume phase transition (VPT) because water is rapidly pulled out from a collapsing polymer network and in result significant reduction of the hydrogel size is observed.

In spite of many experimental works on thermo-sensitive polymers (including PVME), some fundamental problems have still been actual for their aqueous solutions and hydrogels [4, 7, 8]. The main ones relate to: (i) the role of weak interactions (van der Waals and dipole interactions between water and hydrophobic parts of polymer and polymer-polymer interactions) in thermal stabilization of thermo-responsive hydrogels, (ii) the differences in dynamics between water molecules directly interacting to polymer chain (according to Maeda’s model [10] called first- and second-order water) and those consisting of bulk water. Both mentioned problems are crucial from a practical point of view — stability of the polymer chains in water determines the LCST value, while the water dynamics influences the dynamics of VPT [4, 7, 10].

The biggest challenge relates to the fact that redistribution of intermolecular interactions corresponds mainly with molecular rotations and vibrations, while the dynamics of VPT is governed by diffusion. Moreover, the great distinction between the size of water molecule and polymer chain results in significant difference in their mobility. Thus, extremely different size and time scales should be taken into account to simulate hydrogels properly.

In this work the main idea of a graining procedure of PVME-water system and its implementation to study aqueous solution of linear PVME is shown. One of the crucial aims is to distinguish differences in dynamics of water molecules at various states (bulk as well as water interacting with polymer chains). The dynamics of low molecular weight solvent (water) in polymer solution in the athermal case and energy of interactions between solution ingredients were investigated. Monte Carlo (MC) method was used to study excluded volume interaction, while quantum-mechanical (QM) calculations served as a tool to describe electrostatic interactions. This choice was dictated by the area of applicability of both methods. It is worthy to note that there is no experimental technique useful to resolve the mentioned problem. Calculations are discussed in light of experimental data obtained by fluorescence correlation spectroscopy.

## Methods

### Quantum mechanics calculations

To characterize thermal intermolecular interactions in PVME-water system the *ab initio* Møller-Plesset second correction method (MP2), based on *Schrödinger’s equation* [11], was used. Various PVME-water complexes differing in composition were tested. Calculations were performed with Gaussian 09 [12] software. The MP2 method was chosen because it does not introduce too many simplifications. Thus, different structures and interactions in complex systems may be studied without any initial assumptions.

Different computational variants were performed in the 6-31G(d,p) basis set [13]. The following strategy was implemented for all investigated systems to compute energies of intermolecular interactions:

1. Structure optimization was performed in vacuum.
2. The final results were additionally corrected by the basis set superposition error (BSSE) [14].

To check the correctness of applied method water dimer was examined in vacuum (*E*_*int*_ = −29.49 kJ mol^-1^) and in a reaction field using the integral equation formalism model for water (IEFPCM) [15] (*E*_*int*_ = −23.97 kJ mol^-1^ for MP2). It appeared, that both *E*_*int*_ values were overestimated in comparison to real value (−21 kJ mol^-1^ [16]). The best approximation of experimental value of *E*_*int*_ in water was obtained by structure optimization with BSSE correction (*E*_*int*_ = −21.22 kJ mol^-1^).

### Dynamic lattice liquid model — cooperative dynamics

In presented Monte Carlo (MC) studies cooperative dynamics was used. The only one model operating in a fully dense system (density factor equal to 1) with proper dynamics is the dynamic lattice liquid (DLL) model [17]. This model has already been successfully applied to investigate many non-equilibrium physical phenomena including diffusion limited aggregation [18], reaction–diffusion fronts propagation [19], dynamics of linear [20] as well as cross-linked polymer systems [21], spinodal decomposition [22], and diffusion in crowded environments [23].

**Fig. 2:**
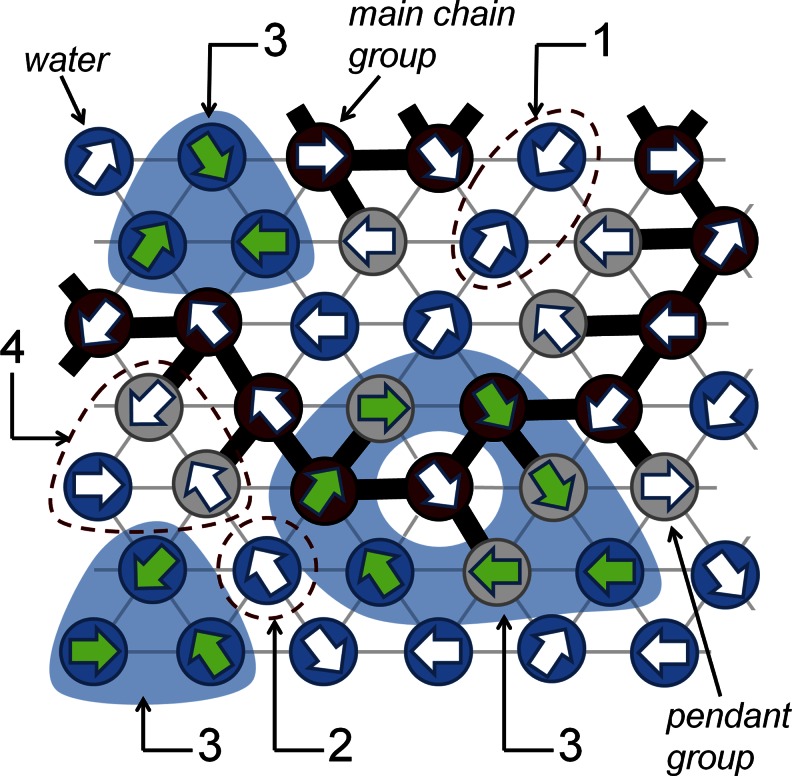
Water-PVME system (see also Fig. 5) with DLL model dynamics illustrated on 2D triangular lattice. Numbers indicate different local movement scenarios (see the text for details)

In the DLL model, the molecular system is considered as an assembly of structureless beads representing atoms or groups of atoms. The model permits simulation of dense complex systems with all lattice sites occupied (no holes of molecular size). The DLL model satisfies the continuity and excluded volume conditions (also for bonds) [17]. Diffusion movement attempts of every bead are represented by a randomly selected unit vector, pointing to a neighboring lattice site (direction of attempted motion). All vectors representing non-successful attempts (an attempt that violates the exclude volume — Fig. 2 (1), creates a vacant site — Fig. 2 (2) or breaks a bond — Fig. 2 (4)) are set to zero and the molecules remain in their hitherto occupied positions. Consequently, only the beads participating in correlated sequences (Fig. 2 (3)) are not immobilized (successful movement attempts), i.e., only vectors contributing to self-avoiding closed paths (called loops) remain.

Molecular displacements are performed by shifting beads along the closed loops — each bead to a neighboring lattice site, according to vector of displacement. In the long time limit DLL dynamics leads to a Brownian walk trajectory, for every molecule, with jumps distributed randomly in time [20]. Practical implementation of the DLL model for complex liquid consists of a few main steps (definition of one time unit called Monte Carlo Step — 1 MCS):

- generation of random vector field,
- elimination of all attempts not satisfying the continuity condition, leaving only closed loops indicating paths of possible successful rearrangements,
- elimination of loops where a bond would be broken or bond would be jumped over,
- performing an energetic test if thermal interaction is included,
- displacement of molecules along closed paths to the neighboring sites (each molecule replaces its neighbor).

Simulations were carried out on 50^3^ FCC lattice with periodic boundary conditions. This system size is large enough to minimize the finite size effect on diffusion [24]. The results were averaged, for times below 10^5^ MCS, over 30 independent runs for each sample. Molecules interacted only by their excluded volume and bonds. Presented herein results concern the athermal case.

## Results

### Electrostatic interactions — QM calculations

The structure optimization as well as an energy of PVME macromolecule was essential to estimate the strength of its interactions with water. The ideal solution to calculate the PVME energy should take into account the macromolecules with length of c.a. 1000 monomer units (what corresponds to real size of experimentally studied systems [21]). Then, all effects relating to conformational changes, steric hindrance, and intrachain interaction may be modeled. Obviously, such a length scale is currently inaccessible for quantum calculations. An usage of PVME tetramer, pentamer, and higher oligomers explicitly interacting with water resulted in non-converged calculations. Thus, the PVME monomer was used to study intermolecular potentials and trimer was chosen to model PVME-water complexes.

**Fig. 3:**
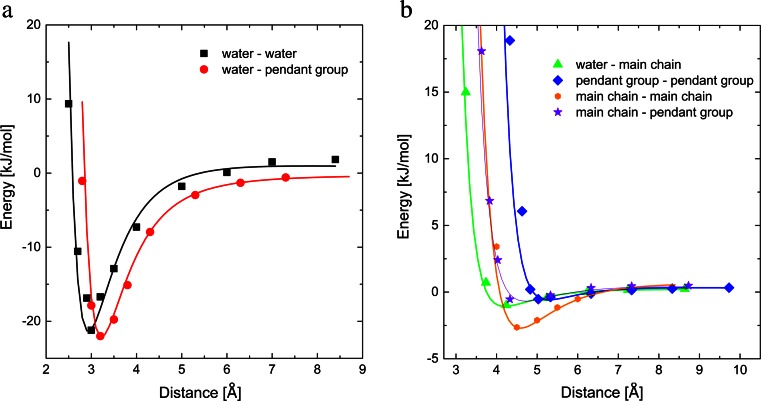
Potentials of **a** strong interactions (water-water, water-pendant group) and **b** weak interactions (water-main chain, main chain-main chain, main chain-pendant group, group-pendant pendant group) in PVME-water system

Proposed graining procedure (see Coarse graining procedure for MC calculations and sample preparation) results in six fundamental interactions in PVME-water system: water-water, main chain-water, pendant group-water, main chain-main chain, main chain-pendant group, pendant group-pendant group. To characterize mentioned interactions different complexes were studied with different distances between molecules. The results are presented in Fig. 3. The energies relating to the particular interaction were calculated according to the general formula (1):

1 $$ {E}_{int}=\left({E}_i+{E}_j\right)-{E}_{ij}, $$

where *E*_*i*_ and *E*_*j*_ — energy of water or optimized PVME monomer or trimer molecule with the appropriate setting, *E*_*ij*_ — energy of the suitable complex. Indexes correspond to the interacting components, according to grain types.

To model properly the real polymer systems the long distance interactions should be taken under consideration. Thus, the changes in energy of various, mentioned above, types of interactions with distance were examined. Performed calculations for longer distances and positive values of energy required an use of single point (SP) command without optimization procedure. To improve obtained values of energy the BSSE correction in MP2 method was applied. This approach is a simple way to answer the question, how does an energy of interaction depend on distance between interacting species.

Figure 3 presents the potential curves for various intermolecular interactions in PVME-water system of the distance between center of coarse grained group of atoms (see Coarse graining procedure for MC calculations and sample preparation). All data were fitted using the Lenard-Jones and electrostatic potentials [25] expressed as follows:

2 $$ {E}_{int}=\frac{D_{ij}}{r_{ij}}+{C}_{ij}\cdot \left[\frac{A_{ij}}{r_{ij}^9}-\frac{B_{ij}}{r_{ij}^6}\right], $$

where the first term *D*_*ij*_/*r*_*ij*_ describes Coulombic interactions and the second one *C*_*ij*_ · [*A*_*ij*_/*r*_*ij*_−*B*_*ij*_/*r*_*ij*_] expresses Leonard-Jones 9–6 function.

Obtained results (see Fig. 3) allowed to classify intermolecular interactions in the investigated system into two groups:

- strong interactions (water-water and water-pendant –O–CH_3_ group) exhibiting deep potential well with minimum for intermolecular distance equal to 3 Å and 3.2 Å,
- weak interactions (main chain-water, main chain-main chain, main chain-pendant group, pendant group-pendant group) with the shallow potential well and the equilibrium distance varied between 4–5 Å.

The similar shape of the potential wells for both types of strong interactions suggests that the equilibrium state in aqueous solution of PVME is metastable, as it is impossible to point at a preferable configuration - water molecule interacts with the same force with PVME as well as with other water molecules. The strength of the other interactions is at least ten times lower and due to that, they may be neglected in further QM considerations.

**Fig. 4:**
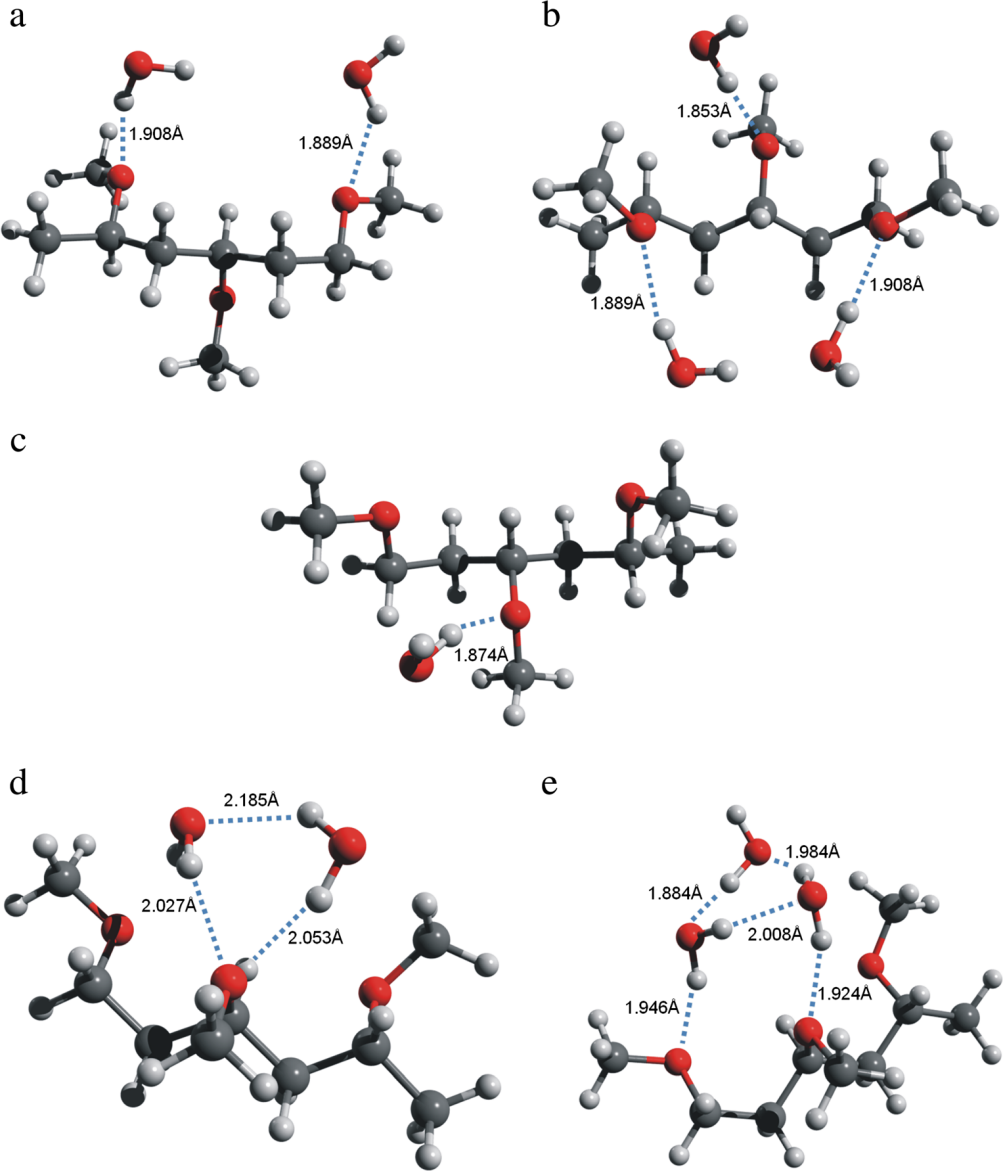
Optimized structures of: **a**-**c** PVME trimer/water systems with component ratio 1:1, 1:2, and 1:3 respectively, **d**-**e** PVME trimer/water 1:2* and 1:3* system with water forming molecular clusters. The formed hydrogen bonds are marked with dotted lines. Numbers present H-bond distance

In the next step, the hydrated PVME trimer with various number and arrangement of water molecules (complexes) was analyzed. To test an influence of water clustering on water-polymer interactions two types of PVME-water complexes were investigated. In the first type a presence of a single water molecule in the neighborhood of particular pendant group was assumed (optimized structures are presented in Fig. 4a and b), while in the second one, water clusters are considered (Fig. 4d and e). In Fig. 4c the optimized structure of water-PVME complex with one water molecule is shown for comparison.

The total interaction energy between PVME trimer and water was calculated according to the following formula:

3 $$ {E}_{int}=\left({E}_P+n{E}_W\right)-{E}_{PW}, $$

where *E*_*P*_ — energy of optimized PVME trimer, *n* — number of water molecules in the system, *E*_*PW*_ — energy of PVME/*n* — water systems.

Comparison of the obtained results for PVME-H_2_O 1:1, 1:2, and 1:3 systems, where PVME trimer was combined successively with 1, 2, and 3 water molecules, has been presented in Table 1. The interaction energies are additive only in these cases. The other two configurations (1:2* and 1:3*) should be considered separately. Figure 4 evidently showed that in these cases molecules form cyclic structures. According to Xantheas [26], the energies of cyclic clusters are lower in comparison to linear ones. It means that obtained *E*_*int*_ values should be lower than the predicted ones. The reverse effect results from the lengthening of the water-water and water-pendant group hydrogen bonds in the cyclic structures.

**Table 1:** Energies of H-bonds optimized for various PVME trimer/water systems in MP2/6-31G(d,p). Predicted energy corresponds to the sum of the suitable number of water-water and water-pendant group interactions

Reference	PVME-H_2_O Composition	*E*_*int*_ [kJ mol^-1^]	Number of H-bonds		Predicted energy [kJ mol^-1^]
			water – water	water – pendant group	
Fig. 4c	1:1	−29.33	–	1	–
Fig. 4a	1:2	−58.43	–	2	−58.66
Fig. 4b	1:3	−87.37	–	3	−87.99
Fig. 4d	1:2*	−55.27	1	2	−76.49
Fig. 4e	1:3*	−101.92	3	2	−117.18

### Coarse graining procedure for MC calculations and sample preparation

Polymer materials consist of macromolecules built of thousands or even millions of atoms. Quantum chemistry offers a precise description of intermolecular interactions but only for relatively small systems. It means that only local properties of the whole system may be characterized. In order to properly describe larger systems (such as polymers) the conformational aspects, as well as inter- and intrachain interactions should be taken into account. This requires accessibility to significantly higher time and size scales than those currently offered, even by supercomputers. Contrarily to quantum mechanics, molecular dynamics (MD) [27] or Monte Carlo [28] methods give the possibility to study large systems containing even over 10^6^ elements, but they are dedicated to study macroscopic properties and the molecular details wear away. Thus, the simple passage between these two groups of computational methods is not trivial.

Force field approach works at the atomic level by neglecting the electron–electron and the electron-nucleus interactions. Electronic degrees of freedom are incorporated in empirical potentials for the bond lengths, bond angles, torsion angles, and non-bonded interactions between atoms of different molecules. Even with such approximations the number of chains has still been insufficient to regard the system as a real polymer system. For example, Capponi [25] presented MD research on seven PVME chains consisting of 100 monomers (7014 atoms) in the temperature range 300–400 K, but only static properties of the system were analyzed and no solvent was present in that case. For large-scale problems MC methods seem to be more suitable. In addition, water solvent can be introduced. MC methods are faster than MD but have more simplifications. Typically, studies are conducted on a lattice containing grains in each node. Grains may represent ions, atoms, groups of atoms (called superatoms) or others.

**Fig. 5:**
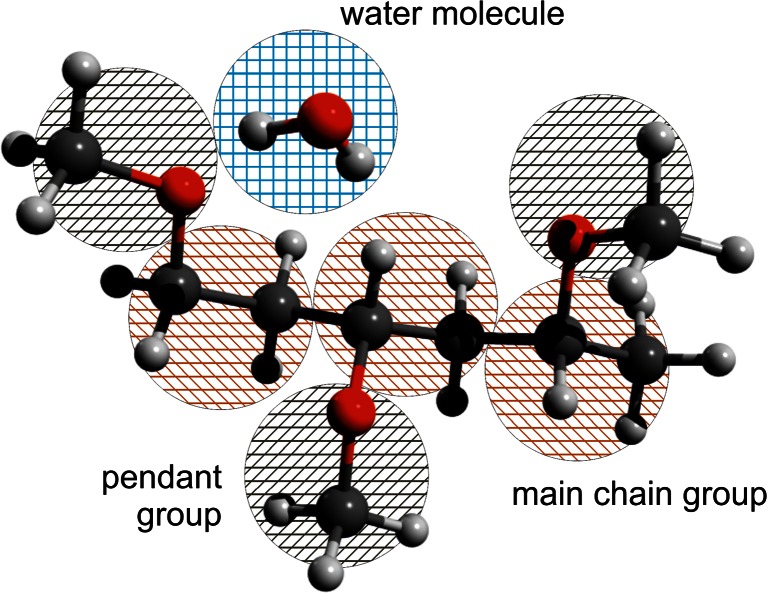
Scheme of the graining procedure applied to describe PVME-water system

The repeating unit of PVME consists of a pendant –O–CH_3_ group connected to –CH–CH_2_– main chain (Fig. 1). The length of C–C and C–O bonds are ca. 1.5 Å, while the length between repeating units is close to 2.8–3.0 Å. This value only slightly depends on polymer conformation. Very similar length characterizes the PVME pendant group. If one considers water molecules placed near polymer chain, it turned out that the distance between water molecule, connected to pendant group by hydrogen bond is also equal c.a. 3 Å. The same average value of intermolecular distances was also obtained for bulk water. Thus, the real PVME-water system can be relatively easily transferred into the network model with lattice constant close to 3 Å, which was assumed as the size of individual grain corresponding to: (i) –CH–CH_2_ a basic main chain fragment called “main group”, (ii) –O–CH_3_ “pendant group”, or (iii) H_2_O molecule (see Fig. 5).

In the case of chain structure, water-water and water-polymer equilibrium distance corresponds to lattice constant reflecting natural length scale close to 3 Å. However, in the case of inter- and intramolecular polymer-polymer interactions equilibrium distance is in the range of 4–5 Å, but the minima of potential wells are so broad and shallow that the effects relating to these types of interactions may be neglected.

Several different samples of aqueous solution of linear PVME with different weight fraction of polymer were virtually prepared to investigate solvent (water) dynamics in water-PVME system. PVME chains were obtained using a well known polymerization scheme [29]. This procedure assures that after polymerization polymer chains are well relaxed and no further relaxation is needed. At the start, a random mixture of assumed molar content of PVME monomers [M_0_], initiators [I_0_], and solvent molecules [S_0_] were placed in network nodes. Monomers were presented as two neighboring lattice nodes — illustrating main chain group and pendant group — connected by unbreakable and inflexible bond. Main chain group was able to create two new unbreakable (irreversible reaction) and inflexible bonds with other monomers (two functional species). Chain propagation was always started from initiator molecule — similar to a monomer molecule able to create a new bond, but only one. Solvent molecules were not able to react and were introduced into the reacting mixture for better representation of a real polymerization experiment. All lattice sites were occupied, i.e., no vacancies were present. Neither termination nor chain transfer reaction were considered and the reactivity of functional groups was set constant and independent of chain length. In all samples the following initial molar contents of particular components were used: [M_0_] =100, [S_0_] =100 and 50 for the most dense sample, [I_0_] varied in range 0.5 ÷ 0.03 to obtain different % wt. of PVME in solution.

The polymerization process was performed on FCC lattice with periodic boundary conditions. All elements were able to move with dynamics described in the next section. The polymerization reaction was stopped when assumed targeted polymer molar content was achieved. Next, all unreacted monomers were replaced by water molecules.

**Fig. 6:**
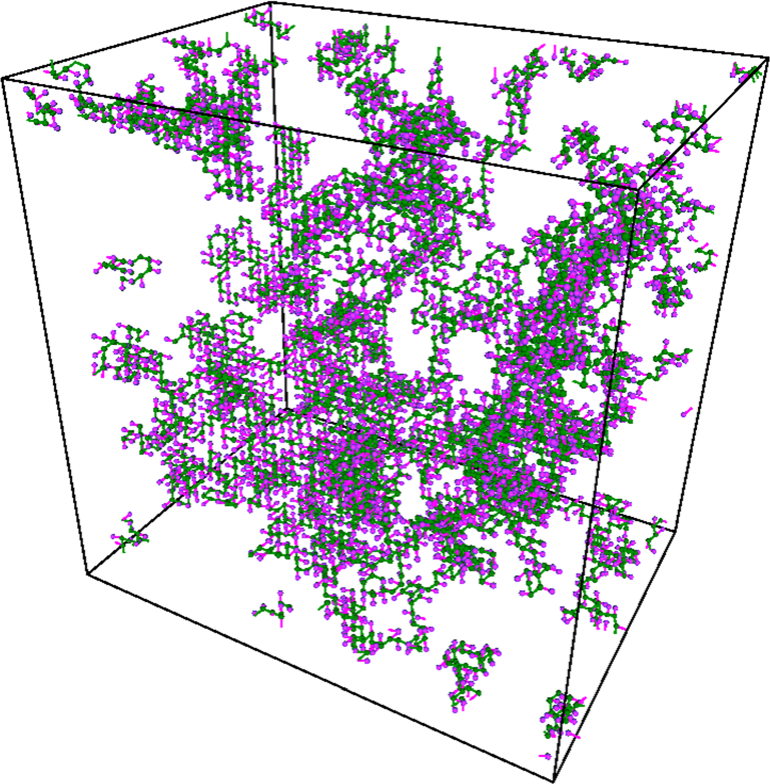
Visualization of investigated system for 10% wt. PVME. Dark color represents main chain groups, light color — pendant groups. Water molecules were not shown for clarity

**Fig. 7:**
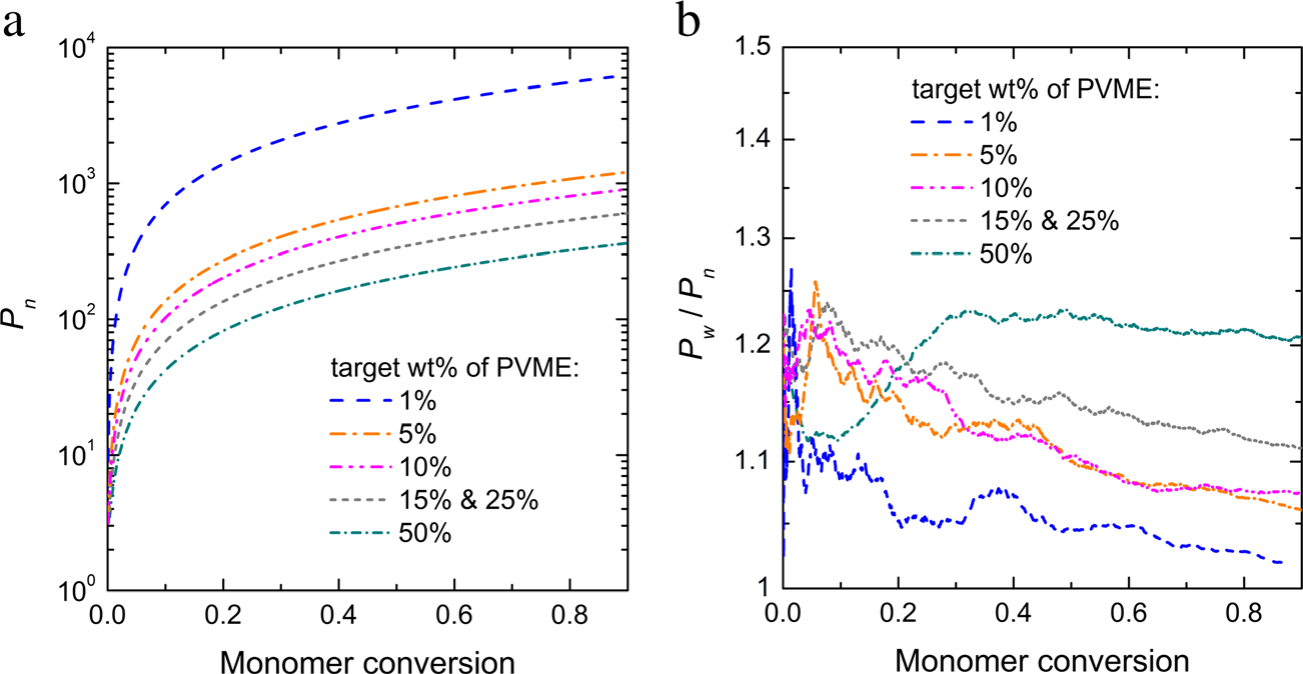
**a** Number average degree of polymerization of all macromolecules as a function of monomer conversion; **b** dispersity as a function of monomer conversion for system used to generate different samples

Several samples with different ratio of polymer molar content to water were generated, see Table 2, where the average chain weight and the total chain number for each sample are also shown. For all samples dispersity varied in the range of 1.20 ÷ 1.25. Prepared samples served as starting configuration for proper simulation of water dynamics in water-PVME system. An example of obtained system configuration for 10% wt. is shown in Fig. 6. Figure 7 shows the number average degree of polymerization (*P*_*n*_) of all macromolecules and dispersity as a function of monomer conversion for systems used to generate particular samples. To illustrate kinetics of the whole polymerization process, the reaction in that case was not stopped at target molar content polymer to water, e.g., sample 50% of PVME was obtained stopping the reaction at monomer conversion close to 0.5. For other samples this value was even lower — for 1% of PVME it was significantly below 0.1. *P*_*n*_ function behaved typically for not cross-linked systems [21]. Weight average degree of polymerization (*P*_*w*_) was similar to *P*_*n*_ (*P*_*w*_/*P*_*n*_ — not far from unity in the whole range of monomer conversion). Data scatter visible in Fig. 7b is due to large dilution.

**Table 2:** Samples characteristics

Sample no.	Molar content	PVME weight fraction	Average chain weight	Chain number
1	1/319.00	1%	67 ± 19	12
2	1/61.22	5%	62 ± 6	62
3	1/29.00	10%	98 ± 10	83
4	1/18.26	15%	96 ± 10	125
5	1/9.67	25%	165 ± 18	125
6	1/3.22	50%	191 ± 16	249

### Dynamics in PVME-H2O systems

**Fig. 8:**
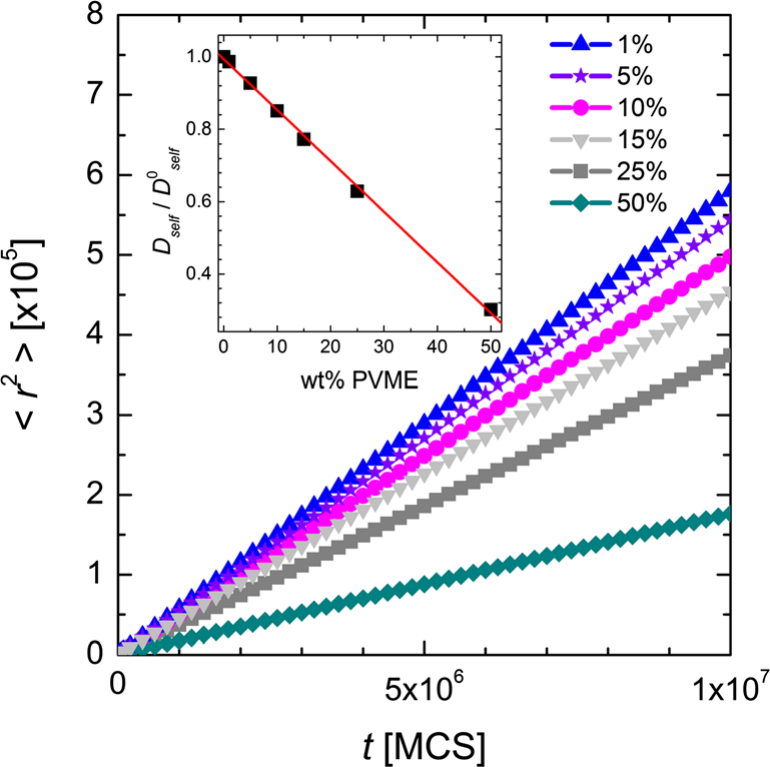
Mean-squared displacement < *r* ^2^ > of water molecules as a function of time for systems with different PVME weight fraction. The inset shows normalized (to pure water) self diffusion constants obtained from Einstein relation

Mean-squared displacement < *r* ^2^ > of water molecules, measured in lattice spacing units, as a function of time *t* in MCS units for different PVME weight fractions is shown in Fig. 8. The values of < *r* ^2^ > increased linearly with time in medium and long time scales. The slope depends on amount of polymer in the systems and it is proportional to self-diffusion coefficient *D*_*self*_ (see Eq. (4)). The inset in Fig. 8 shows normalized (to pure water system *D* ^0^_*self*_) self-diffusion coefficients obtained from Einstein relation for long time scales:

4 $$ <{r}^2>=\frac{1}{N}<{\displaystyle \sum_i{\left[{r}_i(t)-{r}_i(0)\right]}^{\kern0.5em 2}}>=6{D}_{self}t,\kern1em t\to \infty, $$

where *N* is the number of water molecules. Dynamics of water in 1% wt. polymer system is almost the same as in bulk water as evidenced by the ratio close to 1. Increasing PVME concentration slowed down water diffusion. Obtained dependence seems to be linear (at least in investigated range of polymer content) with coefficient of determination 0.999 and the experimental results obtained by fluorescence correlation spectroscopy strongly support it. In this experimental method, the diffusion of fluorescence tracers representing the solvent molecule is investigated. It was clearly shown that the normalized self-diffusion coefficient should linearly depend on the polymer concentration [30].

**Fig. 9:**
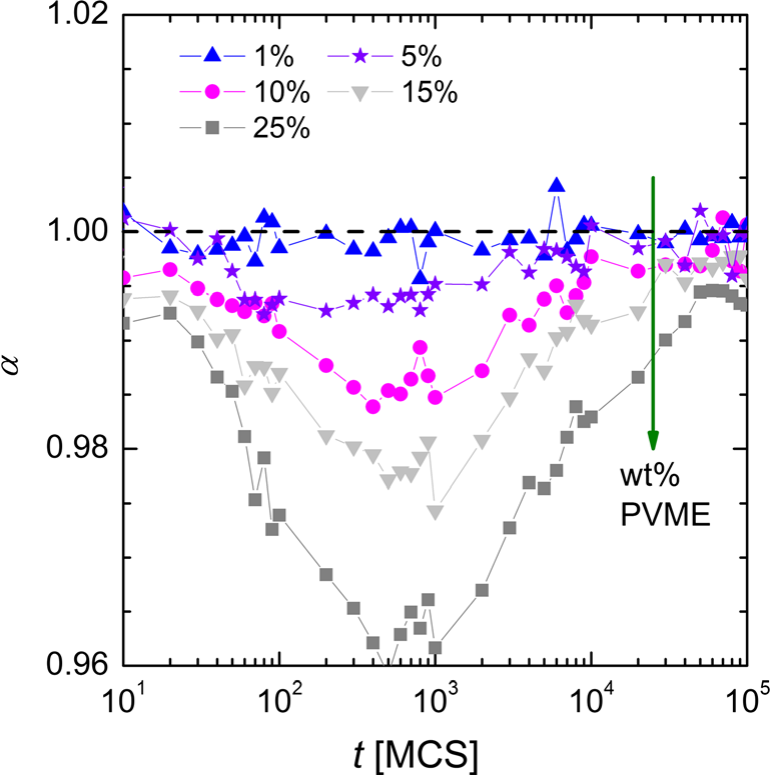
Exponent *α* (slope of < *r* ^2^ > (*t*) functions presented in Fig. 8) as a function of time for different systems with different PVME weight fraction

It is worthy to note, that in short time scales, <*r* ^2^ > (*t*) dependence is not linear when interactions are present in investigated system. In our studies only excluded volume interactions are taken into account. Thus in general, Eq. (4) may be expressed in the form:

5 $$ <{r}^2>\sim {t}^{\alpha },\kern1em \alpha =\frac{\mathrm{d} \log <{r}^2>}{\mathrm{d} \log t}, $$

where exponent *α* indicates the character of the movement. Normal (fickian) diffusion with the Brownian walk trajectory relates to *α ≈* 1, while *α <*1 corresponds to sub-diffusive (slowed-down diffusion) behavior observed in a fractal or porous environment [31]. A convenient way to analyze the time dependent exponent *α* is to plot the logarithmic derivative of < *r* ^2^ > defined as in Eq. (5). The results, averaged over 30 independent runs, are presented in Fig. 9. Changes in the dynamics of solvent can be explained by a different amount of excluded volume from polymer grains and bonds. Low PVME fraction (1 % wt.) does not influence the dynamics of water. When % wt. increases, slowing down is more significant. Slowing down effect manifests for *t * generally lower than 10^5^ MCS and it reaches maximum (minimum of *α* exponent) near *t* = 500 MCS. In longer time scale, normal diffusion is recovered.

**Fig. 10:**
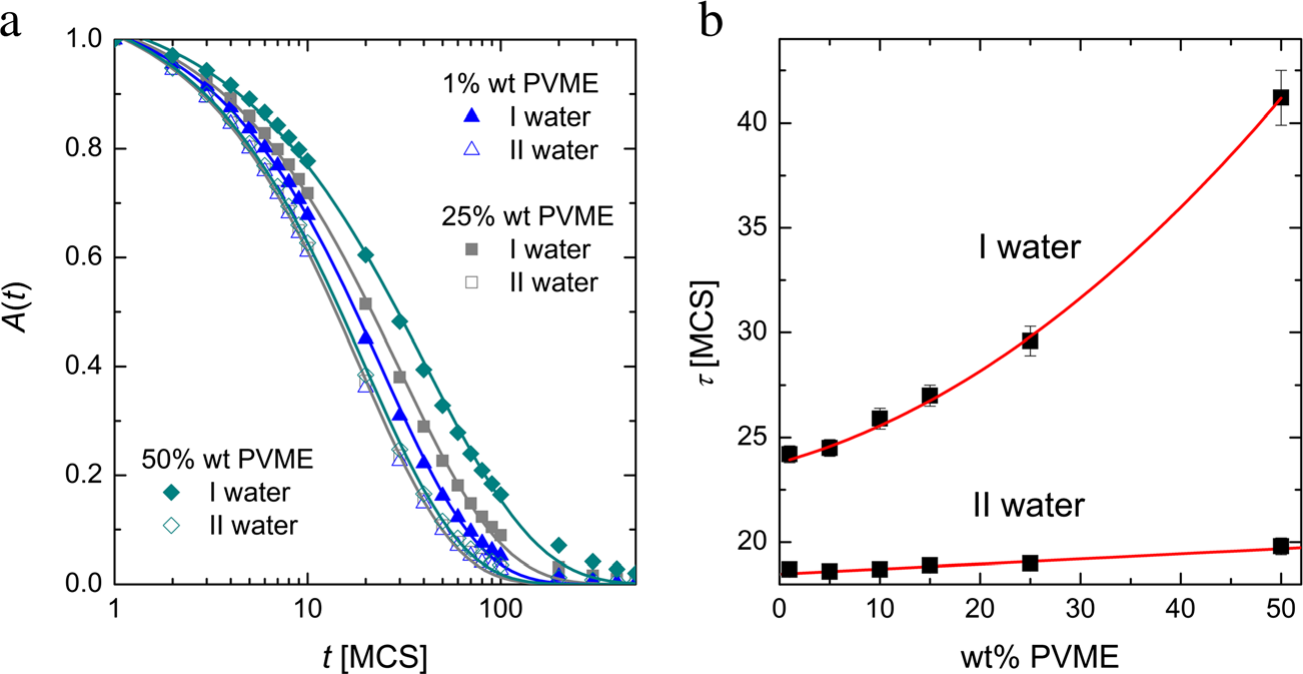
**a** Autocorrelation function for water molecules in two different regions: I water-water in close proximity to polymer groups, II water-water with no polymer in nearest neighbor sites; **b** relaxation times obtained by fitting the KWW function

The relaxation time of water in a different vicinity of polymer is also an interesting property to study. Relaxation time can be obtained using KWW [32] function:

6 $$ A(t)=A \exp \left\{{\left(-\frac{t}{\tau}\right)}^{\beta}\right\}, $$

where *τ* — diffusion relaxation time, *A* — prefactor close to 1, and *β* — fitting parameter, in our case near 0.8. Temporal dependence of autocorrelation function *A*(*t*) was defined as position change of solvent molecules at time *t*:

7 $$ A(t)=\frac{1}{N}{\displaystyle \sum_i{\delta}_i}, $$

where *N* — number of water molecules in analyzed region, *δ* is equal to 1 if the same water molecule occupied site *i* at time *t* and *t* = 0, otherwise *δ* = 0. In Fig. 10a autocorrelation functions are shown, where symbols stands for simulation results (Eq. (7)) and lines present fitting with Eq. (6). To test Maeda’s hypothesis [10] of different water stages in complex polymer systems, two different regions were distinguished: I water — solvent molecules in direct contact with polymer grains, II water — “bulk” water without polymer elements in the nearest neighborhood. Longer relaxation was observed in I water region for higher PVME content. The II water region, as expected, was not noticeably influenced by polymer fraction.

Relaxation times from fitting are shown in Fig. 10b with error bars obtained as regression error. The higher PVME content the longer relaxation time. This reflects the strength of caging effect of water molecules by polymer grains. Relaxation times in II water region are almost independent of polymer concentration. The slight increase is connected with the noticeable influence of I water dynamics (very slow) on II water region when polymer content is high. Distinguishing how the water fractions differ on dynamic behavior in stimuli responsive hydrogels is a key problem, due to the fact, that many phenomena associated with phase separation (crystallization, pre-melting, volume phase transition) are crucial from a practical point of view. All processes listed above relate to the diffusion of water, which, according to presented herein results, is strongly affected by the presence of macromolecules. Thus, the intermolecular interactions should also be introduced to molecular dynamic approach, which will be the next step of our studies.

## Conclusions

In this work the graining procedure of PVME-water system and its implementation to study aqueous solution of linear PVME was shown. Application of MC calculations with usage of cooperative dynamics (dynamic lattice liquid model) allowed to characterize two different fractions of water molecules relating, according to Maeda’s model [10], to bulk as well as to water directly interacting with polymer chain. This observation is extremely important taking into account that there is not any experimental method useful to differentiate between water molecules with normal diffusive properties (bulk water) and the slowed down water molecules (in the nearest neighborhood of polymer). Currently, only the differences in the vibrational and rotational motions of water at different states may be determined experimentally [4, 10].

The quantum-mechanics calculations showed that the formation of cyclic clusters leads to the lengthening of the hydrogen bonds and consequently to higher energies in comparison to linear forms. It is a crucial point looking at an application of QM results to MC calculation considering thermal interactions, as an additivity of interaction energies in PVME-water systems is limited only to simple cases which excluded cyclic structures. The dependences of interaction energy vs. distance for various species showed that the considerations of interactions in MC calculations may be limited only to first coordination shell in a case of strong interactions, while the weak ones may be neglected.
